# *DNA2* in Chromosome Stability and Cell Survival—Is It All about Replication Forks?

**DOI:** 10.3390/ijms22083984

**Published:** 2021-04-13

**Authors:** Jessica J. R. Hudson, Ulrich Rass

**Affiliations:** Genome Damage and Stability Centre, School of Life Sciences, University of Sussex, Falmer, Brighton BN1 9RQ, UK; J.Hudson@sussex.ac.uk

**Keywords:** DNA replication stress, chromosome stability, stalled replication fork, replication fork reversal, chicken-foot structure, replication fork recovery, chromosome underreplication, anaphase bridge, DNA end-resection, Okazaki fragment processing

## Abstract

The conserved nuclease-helicase DNA2 has been linked to mitochondrial myopathy, Seckel syndrome, and cancer. Across species, the protein is indispensable for cell proliferation. On the molecular level, DNA2 has been implicated in DNA double-strand break (DSB) repair, checkpoint activation, Okazaki fragment processing (OFP), and telomere homeostasis. More recently, a critical contribution of DNA2 to the replication stress response and recovery of stalled DNA replication forks (RFs) has emerged. Here, we review the available functional and phenotypic data and propose that the major cellular defects associated with DNA2 dysfunction, and the links that exist with human disease, can be rationalized through the fundamental importance of DNA2-dependent RF recovery to genome duplication. Being a crucial player at stalled RFs, DNA2 is a promising target for anti-cancer therapy aimed at eliminating cancer cells by replication-stress overload.

## 1. Introduction

*DNA2* was first identified in *Saccharomyces cerevisiae* in a screen for candidate DNA replication factors (reflected in the budding yeast name DNA synthesis defective 2) [1,2]. The human homolog is known as DNA replication ATP-dependent helicase/nuclease DNA2 [3,4,5]. Across organisms, *DNA2* is required for cellular proliferation and organismal survival [6,7,8,9]. In human, *DNA2* mutations result in the primordial dwarfism disorder Seckel syndrome [10,11] and have been linked to mitochondrial myopathy [12,13,14]. Depletion of the gene product Dna2 in budding yeast [15] or DNA2 in human cells [16,17] has been demonstrated to result in chromosomal instability.

**Figure 1:**
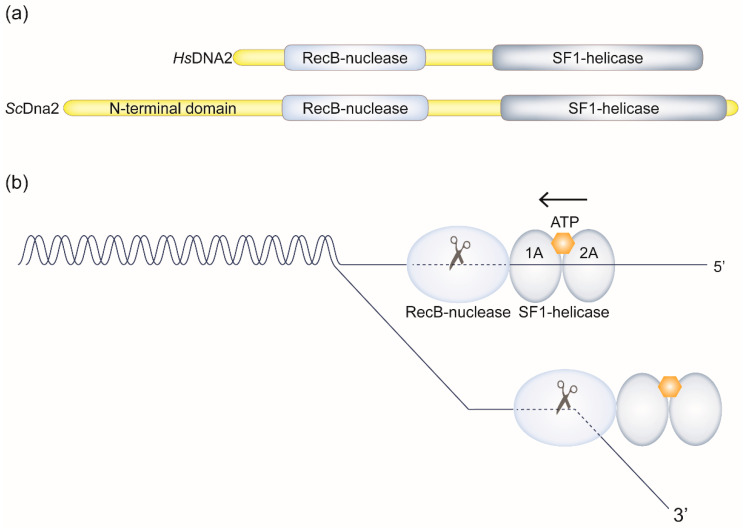
Structure and function of Dna2^DNA2^. (**a**) Across organisms, Dna2^DNA2^ comprises a RecB nuclease and superfamily 1 (SF1) helicase domain. The human (*Hs*) protein is 1060 amino acid residues in length, while budding yeast (*Sc*) Dna2 spans 1522 amino acids with an additional N-terminal domain, which is unstructured and serves a redundant role in S phase checkpoint activation [22]. (**b**) A murine DNA2-ADP-ssDNA structure has been solved [23] showing that ssDNA substrates must thread through an internal channel containing the nuclease active site for cleavage (indicated by a scissors symbol). Exiting this channel, a 5′-ended strand may productively engage the SF1 helicase (represented through its subdomains 1A and 2A with the ATP-binding site indicated), which exhibits 5′-to-3′ translocation polarity (indicated by an arrow). In this arrangement, DNA cleavage will disrupt further translocation activity such that the actions of the nuclease domain effectively control the extent of ATPase/helicase activity. This is consistent with biochemical analyses, in which the full capacity of the helicase activity of Dna2^DNA2^ is only revealed when the nuclease is experimentally inactivated [24,25]. Dna2^DNA2^ can also degrade ssDNA from the 3′-end, but it is unclear whether the helicase domain has a role in this. How the actions of the nuclease and helicase domains are coordinated in vivo remains to be fully elucidated, but a close cooperation of their activities in all pathways involving Dna2^DNA2^ is highly likely.

Dna2^DNA2′^s dual enzymatic activities reside within a RecB-like nuclease domain with single-stranded DNA (ssDNA) specificity and a superfamily 1 helicase domain with 5′-to-3′ translocation polarity [5,6,18,19] (Figure 1). The nuclease and helicase activities of Dna2^DNA2^ are closely linked and have been implicated in multiple DNA replication and DNA repair functions. As we will discuss in detail below, these include Okazaki fragment processing (OFP) during lagging strand DNA synthesis, the processing and recovery of stalled DNA replication forks (RFs), and DNA end-resection during DNA double-strand break (DSB) repair. Each of these processes is important for genome stability, and a key question we will address is which molecular role of Dna2^DNA2^ is essential for cell survival and, by extension, perhaps the most likely explanation for its association with human disease. Importantly, DNA2 has been identified as a candidate for targeted cancer therapy [20,21]. We will discuss the relationship between Dna2^DNA2^′s essential role in cell proliferation and a heightened susceptibility of cancer cells to DNA2 inhibition.

## 2. Discovery of Dna2^DNA2^ and Models for Its Essential Function in Cells

Screening a collection of temperature sensitive *S. cerevisiae* mutants [26] for proteins involved in DNA replication, Campbell and co-workers identified mutant *dna2-1* [1,2]. At the restrictive temperature, *dna2-1* cells (bearing mutation P504S, which maps to the nuclease domain but was later shown to affect Dna2′s nuclease and helicase activities [27]) appeared to quickly cease DNA synthesis, suggesting that Dna2 might be an essential DNA replication factor in eukaryotes [1,2]. While it turned out that Dna2 is not required for DNA synthesis per se, it quickly became apparent that Dna2 is a key replication factor whose dysfunction during S phase of the cell cycle results in DNA damage checkpoint activation and cessation of cell proliferation [28]. To date, two models have been proposed to explain the essential function of Dna2 during DNA replication. According to model 1, Dna2 is indispensable for lagging strand replication, specifically for the removal of DNA flaps at maturing Okazaki fragments [29]. Model 2 suggests that Dna2 is essential for proper processing and recovery of stalled RFs [30]. These models, which are largely based on work in yeast, provide a framework to rationalize the most severe cellular and organismal phenotypes associated with *DNA2* dysfunction.

### 2.1. Model 1: Dna2^DNA2^ Plays an Essential Role during Okazaki Fragment Maturation

During DNA replication, the leading strand is synthesized in continuous fashion by DNA polymerase (Pol) ε, while the lagging strand is discontinuously synthesized by Pol δ in the form of 100–200 nucleotide (nt)-long fragments known as Okazaki fragments [31]. Each Okazaki fragment is initiated by the Pol α-primase complex, which lays down a short primer composed of RNA and initiator DNA. When Pol δ encounters the 5′-end of the preceding Okazaki fragment, this primer is dissociated from the lagging strand template through strand-displacement DNA synthesis and removed with the help of 5′-flap endonuclease Rad27 (FEN1 in human). Rapid nucleosome deposition on the nascent sister chromatids behind moving RFs restricts this process, avoiding excessive strand-displacement synthesis by Pol δ. Accordingly, most Okazaki fragments conform to the nucleosomal repeat length of ~165 bp in *S. cerevisiae* [32]. Once Pol δ dissociates, DNA ligase I fuses adjacent Okazaki fragments to form a mature lagging strand.

#### 2.1.1. The Two-Nuclease OFP Model Involving Rad27^FEN1^ and Dna2^DNA2^

**Figure 2:**
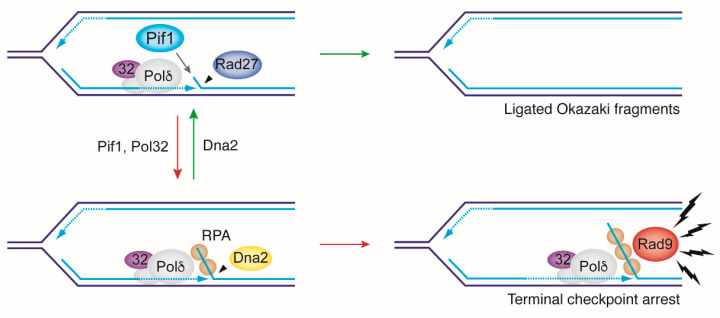
Model for an essential role of Dna2 in OFP. During DNA replication, Pol ε (not shown) synthesizes the leading strand in a continuous fashion while Pol δ synthesizes the lagging strand discontinuously in the form of Okazaki fragments. Limited strand-displacement DNA synthesis by Pol δ and instantaneous cleavage by Rad27 ensures the RNA/initiator DNA primer at the 5′-end of each Okazaki fragment is removed through nick translation (iterative cycles of ~1 nt-flap generation and removal). DNA ligase I seals the final nick to establish the continuity of the lagging strand. The Pol32 subunit of Pol δ and the actions of the Pif1 helicase stimulate the strand-displacement activity of Pol δ, potentially leading to 5′-flaps > ~30 nt, which become refractory to Rad27 by association with ssDNA-binding protein RPA. This creates an essential requirement for Dna2, which, unlike Rad27, is able to cleave RPA-covered flaps. In the absence of Dna2, RPA-covered flaps may accumulate and activate the DNA damage checkpoint in a Rad9-dependent manner, leading to a persistent cell-cycle arrest and, ultimately, cell death.

Dna2 was first implicated in OFP through guilt by association when co-immunoprecipitation experiments indicated a physical interaction with Rad27 [33]. Furthermore, overexpression of Dna2 partially suppressed the lethality of *rad27*Δ cells at elevated temperatures, consistent with a degree of functional compensation. Biochemical studies with Dna2 revealed endonuclease activity on both 5′- and 3′-ended ssDNA, and while free ssDNA ends proved to be the preferred substrate, limited duplex unwinding activity allowed access and some nucleolytic processing at fully annealed DNA ends [18,19]. The presence of ssDNA-binding protein RPA was then shown to stimulate the 5′-flap cleavage activity of Dna2, while it inhibits Rad27 [19,34]. Together, these findings formed the basis for a two-nuclease model for OFP based on cooperation between Dna2^DNA2^ and Rad27^FEN1^ in removing 5′-flaps [19,34,35]. Accordingly, Pol δ-dependent DNA strand displacement during Okazaki fragment synthesis displaces 5′-flaps of sufficient length—i.e., greater than ~30 nt—for RPA to bind. This would both necessitate and stimulate processing by Dna2, which would trim down long 5′-flaps to a size no longer able to support RPA binding, and thus amenable to further processing by Rad27 [36]. Alternatively, Dna2 may fully remove 5′-flaps to allow immediate Okazaki fragment ligation [37] (Figure 2). Dna2′s ability to deal with long, RPA-bound, and short 5′-flaps—as compared to Rad27′s limitation to short flaps free of RPA—could explain why Rad27 is dispensable for cell viability in yeast while Dna2 is strictly required.

Reconstituted OFP reactions, however, have not indicated a significant role for Dna2. In these experiments, the actions of Pol δ and Rad27 are coordinated by the PCNA sliding clamp (to which they both bind), and Rad27 exhibits such great efficiency in cleaving nascent 5′-flaps, generated through strand-displacement synthesis, that flaps large enough to attract RPA are unlikely to emerge [36,38,39]. Further in vitro studies have shown that nascent 5′-flaps act like a molecular brake on Pol δ [40]. As a consequence, OFP proceeds by iterative cycles of single-nt flap formation and instantaneous mini-flap removal by Rad27 [31]. Long-flap formation necessitating intervention by Dna2 does not appear to be an integral part of OFP.

#### 2.1.2. Supporting Genetic Evidence for the Two-Nuclease OFP Model

While OFP reconstitution did not provide concrete evidence of a requirement for Dna2, the identification of three suppressors of *dna2*Δ cell lethality in yeast provided fresh support for an essential role of Dna2 in OFP in vivo. Accordingly, loss (or nuclear exclusion) of the conserved helicase Pif1 [41], the Pol δ subunit Pol32 [41,42,43,44], and the DNA damage checkpoint mediator Rad9 [28,45,46] (see also [47]) was shown to restore viability to *dna2* mutants. These observations indicated that Pif1 and Pol32 drive the accumulation of DNA damage checkpoint-activating DNA intermediates in the absence of Dna2, and that this results in checkpoint-mediated growth inhibition and cell death. Biochemical studies showed that Pif1 and Pol32 promote the processivity of Pol δ, potentially increasing strand displacement on the lagging strand in vivo. Reconstituted Okazaki fragment maturation supported the notion that the presence of Pif1 might result in at least a small proportion of long-flap intermediates [44,48] (Figure 2). In a fully reconstituted OFP reaction, however, Dna2 still was not required for strand maturation, even in the presence of Pif1 and RPA. On the other hand, Dna2 allowed the processing of substrates with pre-formed 30-nt 5′-flaps bound with RPA [49]. Accordingly, Dna2 does provide a way for cells to deal with any eventuality of excessive strand-displacement DNA synthesis during Okazaki fragment maturation (reviewed in [50,51]).

Why cells might have evolved a Pif1-dependent mechanism for long-flap formation at Okazaki fragments, and whether long flaps occur in vivo, remained unclear. Since Pol α lacks proofreading ability, a mechanism for enhanced strand displacement could help sanitize base substitution errors introduced by Pol α on the lagging strand [52]. However, mutation rates of *pol32*Δ and *pif1*Δ strains are very similar to wild type [53], indicating that enhanced strand displacement mediated by Pif1 and Pol32 may not be a requirement for effective removal of Pol α errors from Okazaki fragments. Regardless, considering the sheer number of Okazaki fragments produced in every cell cycle, it is conceivable that the absence of Dna2 might lead to a build-up of RPA-bound 5′-flaps, even if nascent flaps very rarely escape instantaneous Rad27 cleavage, and despite the weak strand-displacement synthesis activity of Pol δ in the face of nascent flaps. RPA-ssDNA is a potent activator of checkpoint kinase Mec1 (ATR in human) [54], and elicits a DNA damage checkpoint response in a dose-dependent manner [55,56]. An accumulation of RPA-ssDNA on the lagging strand could therefore ultimately provoke the lethal event in *dna2*Δ cells: hyperactivation of the DNA damage checkpoint leading to terminal cell-cycle arrest at the G2/M boundary in a Mec1^ATR^ and Rad9 (53BP1 in human)-dependent manner [29,46] (Figure 2).

#### 2.1.3. Limited In Vivo Evidence for an Essential Role of Dna2 in OFP

The use of electron microscopy (EM) has provided some evidence for an accumulation of DNA-flap structures upon loss of Dna2 activity. One study used germinating *Schizosaccharomyces pombe* spores [57], which have the capacity to divide several times before cell-cycle progression is blocked when Dna2 is absent [58]. In the wild type, DNA flaps in the range of ~50 nt were observed near RFs; these structures were more frequent and grew to ~100 to 150 nt when Rad2, the fission yeast Rad27^FEN1^ homolog, and/or Dna2 were absent [57]. This phenotypic similarity between *rad2*^-^ and *dna2*^-^ cells is perhaps surprising, given that Rad2^FEN1^ is believed to process the lion’s share of Okazaki fragment-associated DNA flaps, and that any contribution of Dna2 is likely to be very limited. It remains to be determined whether the DNA flaps observed in *dna2*^-^ germinating spores are dependent on Pif1, which would make them more likely candidates for the toxic intermediates that lead to *dna2*^-^ inviability. Another EM study has identified a range of DNA intermediates that accumulate in replicating budding yeast cells upon acute depletion of Dna2 [59]. The bulk of these structures consisted of double-stranded DNA (dsDNA) molecules with an extended branch of ssDNA with a mean length of ~3000 nt. The potential for extensive RPA binding and the dependence of these structures on Pif1 is consistent with the notion that these unusual intermediates represent the origin of checkpoint activation that restricts the viability of *dna2*Δ cells [59]. On the other hand, rapid nucleosome deposition on nascent sister chromatids is known to block any extended strand-displacement DNA synthesis by Pol δ, making it unlikely that such long tracts of ssDNA are generated by lagging-strand replication [32,60]. This raises the possibility that Pif1-dependent ssDNA accumulation in the absence of Dna2 results from a process other than Okazaki fragment maturation, potentially inadequate RF recovery (as discussed in detail below in Section 2.2).

In human cells, depletion of DNA2 had no measurable effect on the maturation kinetics of Okazaki fragments [16]. This is in contrast to depletion of DNA ligase I or FEN1, which resulted in unligated nascent DNA, indicating failed Okazaki fragment maturation. Importantly, co-depletion of FEN1 and DNA2 did not exacerbate the maturation defect detected upon single depletion of FEN1 [16]. These findings are incongruent with a conserved role of Dna2^DNA2^ in preserving cell viability in absence of Rad27^FEN1^ through functional compensation during OFP [16]. More recently, careful analysis of lagging-strand replication by Okazaki-fragment sequencing in yeast showed that the loss of Okazaki fragment maturation nucleases Rad27 and Exo1, but not Dna2, resulted in reduced—rather than excessive—stand-displacement synthesis by Pol δ [61]. This is in keeping with the above-mentioned inhibition of Pol δ-dependent nucleotide incorporation by nascent 5′-flaps, which restricts flaps at Okazaki fragment junctions to a length of one or a few nucleotides [40,62]. Furthermore, Dna2 did not affect the position of Okazaki fragment termini, even in the absence of Rad27 and Exo1, indicating Dna2 is dispensable for OFP in vivo [61]. Okazaki-fragment sequencing further revealed that while Pif1 stimulates Pol δ progression in vivo, it does so redundantly with Rrm3, a related helicase present in budding yeast [63]. As a consequence, loss of Pif1 did not lead to detectable changes in strand-displacement DNA synthesis. Since reduced strand displacement in absence of Pif1 is the proposed mechanism suppressing *dna2*Δ lethality, this raises serious doubts whether Pif1-mediated toxicity in *dna2*Δ can be explained on the basis of long-flap formation during OFP [63].

In conclusion, Dna2 has the ability to cleave RPA-covered DNA flaps that are refractory to Rad27, supporting the model of an essential role of *DNA2* in policing long-flap formation during Okazaki fragment maturation (Figure 2). Reconstitution of lagging strand DNA synthesis suggests that the strand-displacement activity of Pol δ is limited, and that the odds are greatly stacked against long-flap formation. This resonates with Okazaki fragment sequencing results, which have failed to detect a requirement for Dna2 during OFP in vivo. A minor involvement of Dna2 in Okazaki fragment maturation in vivo is a likely possibility, but other molecular processes must be considered to explain the essential contribution of *DNA2* to cell viability.

### 2.2. Model 2: DNA2 Plays an Essential Role in the Processing and Recovery of Stalled RFs

**Figure 3:**
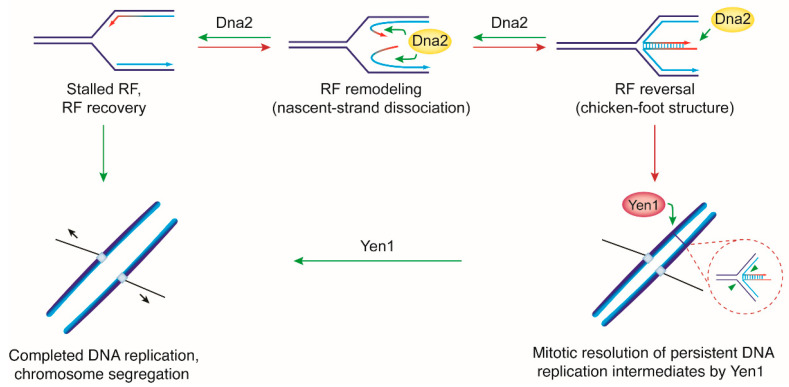
Model for a conserved role of Dna2^DNA2^ in replication fork (RF) recovery. Stalled RFs may be able to resume DNA synthesis and/or remain in a configuration conducive to passive rescue by fusion with an oncoming, active RF. Alternatively, RF remodeling can occur, which requires dissociation of the nascent leading and lagging strands. If the dissociated nascent strands anneal, RF reversal results in a four-way chicken-foot structure. Dna2 has been implicated in the processing of dissociated single-strands and fully reversed RFs. Controlled nascent-strand degradation by Dna2 restores a canonical three-way RF architecture and promotes replication completion. Intermediates escaping the attention of Dna2 physically link sister chromatids at sites of underreplication and preclude chromosome segregation. Holliday junction resolvase Yen1 provides a fail-safe mechanism for the removal of persistent replication intermediates in mitosis. The interplay between DNA2 and the human Yen1-homolog GEN1 remains to be investigated. DNA intermediates colored red are non-productive in DNA replication and their processing (green arrows) is required for complete chromosome replication and proper chromosome segregation.

DNA replication relies on the unfettered activity of RFs, which must track the entire length of every chromosome to synthesize a complete copy of the genome in every cell cycle. Along the way, RF movement can be impaired by DNA damage, ongoing transcription, template-bound proteins, DNA secondary structures, and imbalances in replication factors or deoxyribonucleotide triphosphate (dNTP) pools, which results in replication stress [64]. When RFs stall, they undergo controlled nucleolytic processing and structural rearrangements to promote RF recovery. A prominent pathway involves dissociation of the nascent leading and lagging strands, which may subsequently anneal with one another to convert a three-way RF into a four-way Holliday junction-like structure known as a “chicken-foot” intermediate (see Figure 3). Such reversed RFs have been directly visualized in a range of organisms from yeast to human by EM [65]. RF architectural rearrangements are thought to provide a platform for stable RF pausing, RF reactivation, and homologous recombination-mediated replication restart [66]. Normally transient, stalled and reversed RFs have been shown to accumulate upon loss or depletion of Dna2^DNA2^, implicating the protein in the processing of stalled RF intermediates [59,67,68].

#### 2.2.1. The Dna2 Nuclease and Helicase Activities Promote the Recovery of Stalled RFs

An early indication for the involvement of Dna2 in RF recovery came from *dna2* alleles in budding yeast bearing mutations in the Dna2 helicase domain [45]. In contrast to the nuclease activity, the helicase activity turned out to be non-essential, but Dna2 helicase mutants exhibited sensitivity to DNA base damage, dNTP shortage, and genetic perturbation of the replication machinery. Accordingly, helicase-defective *dna2* cells showed sensitivity to alkylating agent methyl methanesulfonate and did not tolerate the loss of Ctf4 (AND-1 in human), an integral but non-essential component of the replication machinery [45]. Similarly, mutations in the Dna2 helicase domain sensitized cells to the ribonucleotide reductase inhibitor hydroxyurea (lowering dNTP availability) and mutation of another replication factor, Mcm10 [69,70]. These observations of replication stress sensitivity led to the suggestion that Dna2 may be required to overcome RF pausing or participate in the repair of stalled RFs [69]. Since then, a growing list of negative genetic interactions with factors that play critical roles in maintaining the stability of stalled RFs has solidified this notion [43,71]. For example, the most severe negative interactors of helicase-dead allele *dna2-2* were found to include all components of the Mrc1-Csm3-Tof1 (Claspin-Timeless-Tipin in human) fork protection and the Rtt101-Mms1-Mms22 (CUL4-DDB1-MMS22L) fork repair complexes [71].

The first mechanistic insights into how Dna2 might act to facilitate RF recovery came from a study in fission yeast [67]. Dna2 was identified as a binder of chromatin and model RFs when isolated from cells exposed to replication stress. These associations were dependent upon phosphorylation of Dna2 amino acid residue S220, mediated by the S phase checkpoint kinase Cds1 (analogous to Rad53 in budding yeast and CHK1 in human). Loss of Cds1, mutation of the S220 phosphorylation site within Dna2, or inactivation of Dna2 led to an accumulation of reversed RFs detected by EM, in particular upon exposure of cells to replication stress [67]. This was reminiscent of budding yeast, where Rad53 had been show to protect stalled RFs and suppress the accumulation of chicken-foot structures to levels detectable by EM [72,73], and suggested that the checkpoint acts through Dna2 to promote RF recovery. Notably, acute depletion of Dna2 in budding yeast was recently shown to result in an accumulation of aberrant replication intermediates, including chicken-foot structures [59]. In vitro, fission yeast Dna2 was shown to remove 3′-and 5′-ssDNA from DNA three-way junctions mimicking reversed RFs with dissociated leading and lagging strands attached to them [67]. Based on their results, Hu and co-workers proposed the following model: replication stress results in S phase checkpoint-dependent Dna2 phosphorylation and recruitment to stalled RFs. The Dna2 nuclease then degrades any dissociating nascent DNA, essentially counteracting the formation of chicken-foot structures to promote RF stability and recovery (Figure 3). The study did not find a role for Dna2′s helicase activity, but the requirement for the Dna2 helicase activity at stalled RFs was subsequently demonstrated in budding yeast [74,75]. Replication stress-sensitive Dna2 variant R1253Q (encoded by the *dna2-2* allele [45]) was shown to be ATPase/helicase-dead, while retaining wild-type nuclease activity on RPA-covered DNA flaps [74]. Loss of only Dna2′s helicase activity rendered cells unable to fully complete chromosome replication, resulting in anaphase bridge-like structures between segregating chromosomes at mitosis. Viable chromosome segregation became dependent on Holliday junction resolvase Yen1 (GEN1 in human) [76,77,78], a structure-specific nuclease that is activated upon anaphase entry [79,80]. These findings showed that the Dna2 helicase activity contributes to the processing of at least a subset of stalled replication intermediates to ensure DNA replication is faithfully completed before mitosis [74,81]. The helicase activity of Dna2 has been shown to promote ssDNA degradation by the Dna2 nuclease during DSB end-resection [25,82] (see Section 2.3.1), and to facilitate access to, and cleavage of, DNA flaps containing DNA secondary structures [83]. A similar cooperation of the Dna2 helicase and nuclease domains could increase the effectiveness of nascent ssDNA removal from stalled RFs. In an alternative, but not mutually exclusive, scenario, Dna2 may rely on its helicase activity upon chicken-foot formation to dissociate the annealing leading and lagging strands, providing the nuclease domain access for ssDNA degradation. Regardless of the exact mechanism of action, these observations are consistent with coordinated actions of the Dna2 nuclease and helicase activities, at stalled RFs, to maintain or restore fork architectures conducive to replication completion, either by allowing RF restart and/or fusion with an oncoming fork. Occasional replication structures escaping the attention of Dna2 may persist into mitosis and jeopardize proper chromosome segregation, but these can be resolved nucleolytically by Yen1 (Figure 3).

Similar to what has been observed in yeast, human DNA2 appears to be dispensable for bulk DNA synthesis in S phase but required to fully complete chromosome replication. Accordingly, DNA2 depletion in human cells has been shown to lead to chromosome bridges and micronuclei formation at mitosis [16]. More recently, DNA fiber analysis, which allows monitoring replication arrest and restart on the single-molecule level, showed that human DNA2 promotes RF recovery after transient exposure of cells to replication stress induced by hydroxyurea [68] (see Section 3.1). Thus, in contrast to a proposed role in OFP, there is clear evidence that the role of Dna2^DNA2^ in RF recovery is conserved from yeast to human.

#### 2.2.2. Evidence Supporting That Dna2′s Role in RF Recovery Is Essential for Cell Viability

A conserved role of Dna2^DNA2^ in counteracting RF reversal, anaphase-bridge formation, mitotic failure, and replication stress sensitivity raises the question whether RF recovery can account for the essential requirement of Dna2 for cell viability. As mentioned above, in budding yeast, cell death in absence of Dna2 is driven by a Pif1/Pol32-dependent process, which ultimately gives rise to DNA structures that activate a Rad9-dependent checkpoint response, cell-cycle arrest and cell death. Accordingly, deletion/inactivation of *PIF1*, *POL32*, or *RAD9* suppresses *dna2*Δ lethality [28,41,42,43,44,45,46,47]. However, not even the most potent suppressor mutations, those in *PIF1*, restore resistance to exogenous replication stress in *dna2*Δ cells [41,84]. This may suggest that the response of Dna2 to stalled RFs and the toxicity generated along the Pif1/Pol32/Rad9 pathway in unperturbed conditions are separate issues. Conceivably, Pif1/Pol32/Rad9-driven toxicity in *dna2*Δ cells may relate to faulty OFP, and severe replication-stress sensitivity in *dna2*Δ *pif1* double-mutant cells to perturbed RF recovery. In other words, both pathways could in principle make independent contributions to loss of viability in absence of Dna2. However, recent findings indicating that, in *dna2*Δ cells, Pif1-dependent toxicity arises at unresolved RFs as a result of excessive recombination-dependent replication (RDR) restart, suggest otherwise [84]. This link between failed RF recovery in *dna2*Δ cells and Pif1-driven cell death, as explained in more detail below, indicates that Dna2-dependent RF recovery represents not only an important function in promoting replication completion, but also the sought-after essential function provided by Dna2 to ensure cell survival [30,84].

In budding yeast, *dna2*Δ cells that survive on account of the disruption or nuclear exclusion of Pif1 exhibit a chromosome-underreplication phenotype that manifests itself in the form of post-replicative chromosome entanglements [84]. In unperturbed conditions, this phenotype was most prominent for chromosome XII containing the difficult-to-replicate rDNA array, and *dna2*Δ *pif1* double mutants required Yen1 to maintain viability. Following transient RF arrest induced by hydroxyurea, chromosome underreplication was exacerbated and extended genome-wide. Collectively, these results demonstrate that loss of Dna2 compromises proper RF recovery and replication completion, and that these defects are independent of Pif1 [84]. To test the effect of Pif1, Dna2 helicase-dead cells were used. These cells are compromised in RF recovery and replication completion, but less so than *dna2*Δ cells; while they are affected by Pif1-mediated toxicity, they can be grown in the presence of Pif1 [74,84]. When these Dna2 hypomorphic cells were transiently exposed to replication stress, post-replicative DNA damage checkpoint activation and cell-cycle arrest at the G2/M transition ensued in a strictly Pif1/Rad9-dependent manner [74,84]. This is consistent with inappropriate actions of Pif1 at perturbed replication intermediates that would normally be handled by Dna2.

**Figure 4:**
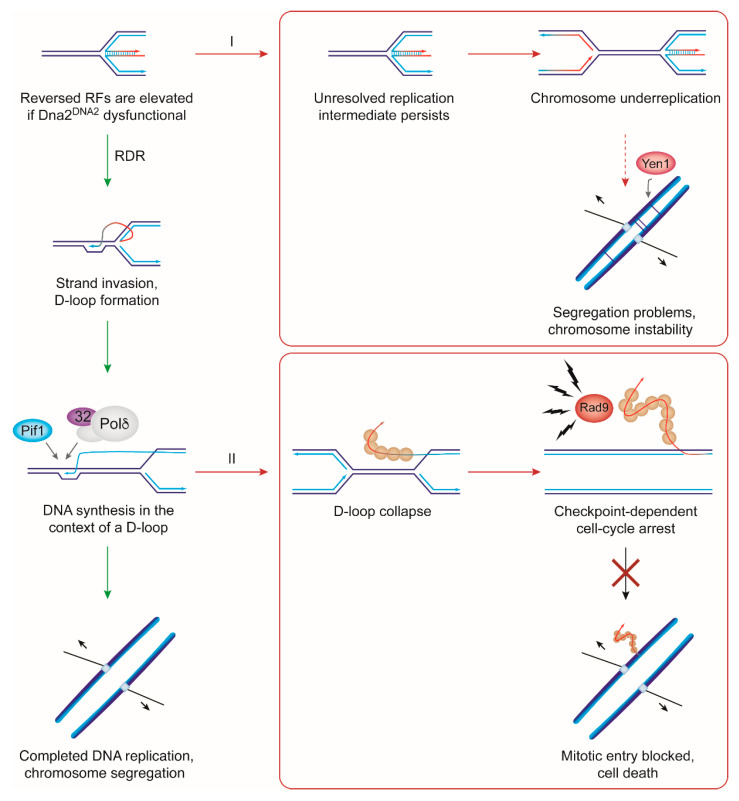
Model explaining why RF recovery by Dna2 is essential for cell viability. If Dna2 does not counteract RF reversal, chicken-foot structures accumulate, causing two problems: (**I**) Unresolved RFs persist, leading to impaired RF convergence and chromosome underreplication. If cells reach mitosis, anaphase bridges form between sister chromatids, resulting in mitotic catastrophe (partially mitigated by Yen1). (**II**) Persisting chicken-foot structures favor recombinational RF recovery by recombination-dependent replication (RDR). This promotes replication completion, but excessive RDR in the absence of Dna2, due to the intrinsic fragility of RDR intermediates, results in ssDNA exposure by way of D-loop collapse. ssDNA tracts may persist after RF convergence, which is potentially reflected in dsDNA with extended ssDNA tracts detected by EM upon Dna2 depletion [59]. RPA-bound ssDNA activates the Rad9-dependent DNA damage checkpoint, precluding cell-cycle progression and causing cell death. The model is supported by findings that Pif1/Rad9-driven toxicity in *dna2* cells maps to Pif1′s role in RDR [84]. Likewise, Pol32 promotes D-loop DNA synthesis [92], and, similarly to Pif1, the absence of Pol32 suppresses the lethality of *dna2*Δ cells. Red DNA strands denote non-productive intermediates.

An important insight into Pif1-mediated toxicity came from the realization that Pif1-driven checkpoint activation in *dna2* cells correlates with Pif1′s ability to support homologous recombination-dependent DNA synthesis [84,85,86,87], a mechanism that can be used to restart stalled or broken RFs [88]. At a broken RF, the ensuing single-ended DSB is resected to expose a 3′-overhang that serves as a strand-invasion substrate for recombinase Rad51. Strand invasion into the intact sister chromatid generates a displacement loop (D-loop), which becomes the site of DNA synthesis in a pathway known as break-induced replication (BIR) [89]. At stalled RFs, fork reversal into a chicken-foot structure provides a recombinogenic, DSB-like substrate at the tip of the regression arm. Following 5′-end resection, Rad51 mediates strand invasion ahead of the backtracked RF to restart DNA synthesis by RDR. D-loop progression during BIR/RDR is strictly dependent on Pif1 (and Pol32) [90,91,92,93,94]. DNA synthesis in the context of a D-loop can proceed over long distances, but D-loop collapse is frequent, resulting in the exposure of long tracts of newly synthesized ssDNA [95,96,97,98,99]. Accordingly, excessive use of RDR in lieu of RF recovery by Dna2 could create a situation where toxic levels of ssDNA are generated through D-loop collapse, providing a plausible explanation for Rad9-dependent checkpoint activation and terminal cell-cycle arrest in Dna2-mutant cells [30,84] (Figure 4). In contrast to OFP, where nascent nucleosomes and flap formation restrict any extended strand-displacement DNA synthesis by Pol δ [32,40,60,62], RDR DNA-synthesis tracts can be thousands of nucleotides in length. A model of excessive RDR and D-loop collapse would therefore predict the accumulation of heterogeneously sized ssDNA tracts of extensive length rather than small flaps in *dna2*Δ cells. Interestingly, Pif1-dependent intermediates consisting of dsDNA with ssDNA branches of a few hundred to more than 10,000 nt have been observed in budding yeast upon depletion of Dna2 [59]. In future, it will be important to address directly the ability of Dna2 to limit RDR at a defined locus of RF arrest.

In conclusion, Dna2′s RF recovery role is of fundamental importance for the completion of DNA replication and to prevent the excessive use of alternative fork restart pathways. Considering the increased complexity of the human genome, a similarly essential requirement of human DNA2 on account of its RF recovery function seems likely (discussed in Section 3 below).

### 2.3. Other Functions of DNA2

In addition to OFP and RF recovery, DNA2 has been implicated in DSB end-resection and the maintenance of telomeric, centromeric, and mitochondrial DNA (mtDNA) across organisms. The yeast-specific N-terminal domain of Dna2, which is not conserved in higher eukaryotes, serves a non-essential role in S phase checkpoint activation through physical interaction with Mec1^ATR^ [22]; for a discussion of this checkpoint stimulatory function, please see [100]. For the purpose of this review, we will briefly describe Dna2′s end-resection activity, as well as its telomeric/centromeric and mitochondrial roles, which could entail specialized Dna2 activities, or reflect the ability of Dna2 to promote the progression of RFs through difficult-to-replicate regions of the genome.

#### 2.3.1. DSB End-Resection

Several nucleases mediate end-resection at chromosomal DSBs, and Dna2^DNA2^ has been shown to be one of them [101,102]. End-resection is a prerequisite for the faithful repair of DSBs by homologous recombination-dependent mechanisms [103]. To initiate recombinational repair, DSBs must be resected at the 5′-terminated strand, which results in 3′-ssDNA overhangs that constitute the substrate for the central recombinase Rad51^RAD51^. Polymerization of Rad51^RAD51^ on the 3′-overhangs generates nucleoprotein filaments capable of seeking out and invading a DSB repair template of homologous DNA sequence. Repair DNA synthesis and the resolution of recombination intermediates then restores the damaged sequence at the break site [103].

DSB end-resection can be divided in two phases including short-range resection dependent on the Mre11-Rad50-Xrs2 (MRE11-RAD50-NBS1 in human) complex and Sae2^CtIP^, followed by long-range resection mediated redundantly along pathways defined by Exo1^EXO1^ and Sgs1 (Bloom syndrome helicase BLM in human)-Dna2^DNA2^ [102,104,105,106]. In contrast to Exo1^EXO1^, which degrades DNA exonucleolytically, Dna2^DNA2^, due to its anatomy, prefers substrates with overhanging ssDNA that can feed into the tunnel-like structure containing the nuclease active site [23] (see Figure 1). The functional integration of Dna2^DNA2^ with Sgs1^BLM^ ensures processive duplex unwinding, which generates these ssDNA overhangs. RPA directs the nucleolytic activity of Dna2^DNA2^ towards the 5′-terminated strands [101,107,108,109]. In human, Werner syndrome helicase WRN can take the place of BLM and collaborate with DNA2 in DSB end-resection [24,110]. The helicase activity of Dna2^DNA2^ plays an accessory role and increases the speed and 5′-strand degradation specificity, during DSB end-resection [25,82]. In the absence of Dna2, Exo1 still provides significant end-resection capacity in budding yeast. Consistent with this, disruption of Dna2-dependent end-resection by deletion of *SGS1* does not result in cell lethality [106]. Thus, while the DSB end-resection function of Dna2^DNA2^ is conserved, the essential role of *DNA2* for cell survival lies elsewhere.

#### 2.3.2. Dna2^DNA2^ and the Maintenance of Telomeric, Centromeric, and mtDNA

Dna2^DNA2^ has been observed at telomeres in yeast and mouse embryonic fibroblasts [8,111]. In budding yeast, loss of Dna2′s helicase activity resulted in abnormal telomere length, indicative of perturbed telomere replication [45,111]. Similarly, DNA2 haploinsufficiency in mouse cells resulted in telomere fragility and loss [8]. Telomere fragility can arise under replication stress conditions as a consequence of incomplete replication [112,113,114]. In DNA2-deficient cells, this phenotype was aggravated in the presence of G-quadruplex DNA (G4)-stabilizing drugs, consistent with a requirement for DNA2 to replicate through DNA secondary structures formed at TTAGGG repeats found within telomeric DNA. This has been suggested to reflect the ability of DNA2 to cleave and remove G4 structures, thereby facilitating RF progression across TTAGGG repeats [8]. Yeast and mouse Dna2^DNA2^ have been shown to cut G4 DNA with a proximal ssDNA tail, for example within a 5′-flap intermediate [8,115]. The mammalian enzyme was much less effective in cleaving G4s in a bubble structure delimited by dsDNA on either side, and unable to cleave substrates mimicking G4 formation within the lagging strand template [8]. These observations are consistent with the structure of mouse DNA2 showing the need of threading a ssDNA end into the tunnel containing the nuclease active site for cleavage [23] (see Figure 1). Accordingly, the ability of DNA2 to directly remove G4s in the parental DNA to promote RF progression in this way is limited. This raises the possibility that replication stress induced by G4 DNA (and potentially by other telomeric features including RNA:DNA hybrids involving telomeric repeat-containing RNA [116]) could be resolved by DNA2 in its capacity as a RF recovery factor. Hence, DNA2 may process replication intermediates to counteract the reversal of RFs stalled at G4 structures and other telomeric obstacles to promote restart and the completion of telomere replication. In other words, loss of the role of Dna2^DNA2^ in RF recovery described above may manifest itself most prominently in difficult-to-replicate areas, such as, for example, telomeric DNA in mouse cells [8], or the rDNA in budding yeast [84]. This resonates with findings of incomplete replication of centromeric regions in human cells upon loss of DNA2 [117]. Cre-mediated excision of *DNA2* in cycling human cells led to shortened replication tracks in centromeric DNA; restoration of normal track length required both the helicase and nuclease activity of DNA2 [117]. Like telomeres, centromeres contain repetitive DNA sequences and pose a particular challenge to the progression of RFs. DNA2 could be needed to remove obstacles arising at centromeres, for example DNA secondary structures, or to ensure recovery of RFs stalled along the centromeric regions of chromosomes. Overall, the exquisite sensitivity of the rDNA, telomeres, and centromeric regions supports the conjecture that counteracting RF arrest and the associated risk of chromosome underreplication is the critical role of Dna2^DNA2^ in ensuring cell viability.

While Dna2^DNA2^ mediates chromosome replication in the nucleus, the protein has also been implicated in mtDNA replication [41,118,119]. Its precise function in mitochondria is among the least-well understood aspects of Dna2^DNA2^ biology, in part because the mode of mtDNA replication has not been fully elucidated [120]. Interestingly, human DNA2 was shown to accumulate on mtDNA under conditions of replication stalling induced by mutations within the mitochondrial replicative helicase Twinkle [119]. This may suggest a similar role of Dna2^DNA2^ in the recovery of stalled RF intermediates in the nucleus and mitochondria.

## 3. An Essential Role of Human DNA2 in RF Recovery?

As described in Section 2, yeast Dna2 has been implicated in DSB end-resection, OFP, and RF recovery. The latter two pathways have each been proposed to account for the essential requirement for *DNA2* [29,30]. Precise measurements of Okazaki fragment maturation in vitro, and recent Okazaki fragment sequencing results in particular, have indicated that the contribution of Dna2 to OFP could be much less significant than previously assumed [40,61]. Experiments in human cells have failed to detect a role for DNA2 in OFP. Duxin and co-workers showed that following replication, nascent DNA did not contain excess unligated Okazaki fragments upon depletion of DNA2, unlike in the absence of FEN1, which led to the suggestion that DNA2 may not be involved in OFP in human [16]. In contrast to OFP, Dna2^DNA2′^s role in RF recovery is conserved from yeast to human and chromosomes remain incompletely replicated when Dna2^DNA2^ is absent or depleted across organisms [8,16,84,117,119].

### 3.1. RF Restart Mediated by Human DNA2

Similar to findings in yeast, human cells depleted for DNA2 are inefficient at restarting stalled RFs. Accordingly, DNA2 depletion results in sensitivity to DNA-damaging agents and dNTP depletion by hydroxyurea, i.e., conditions that impede RF progression [16,17]. DNA2 is also required to survive replication stress associated with oncogene expression [21] (discussed in Section 4.2.1). DNA fiber analysis showed, at the level of individual forks, that DNA2 aids efficient RF restart following treatment of cells with hydroxyurea, the topoisomerase poison camptothecin, or the DNA crosslinking-agent mitomycin C. In this pathway of RF recovery, DNA2 was found to act epistatically with the WRN helicase [68]. Although WRN and BLM have both been shown to function with DNA2 in end-resection at DSBs (see Section 2.3.1), in fork restart BLM cannot compensate for the loss of WRN, potentially reflecting the presence of differing protein complexes or DNA target structures at these distinct repair sites.

DNA2 supports human cell survival not only under conditions of exogenous replication stress, but also in unperturbed growth. The defects observed following DNA2 depletion appear to be caused by a problem in completing DNA replication, suggesting that the role of DNA2 in restarting stalled RFs may be essential under normal growth conditions [16,17,117,119]. Thus, reminiscent of the situation in yeast [74,84], cells traversed S phase and achieved bulk replication following depletion of DNA2, but accumulated in G2 phase and mitosis with associated chromosome shattering, aneuploidy, micronuclei formation, and internuclei DNA bridges. Normal cell proliferation required both the nuclease and helicase activity of DNA2 [16,17].

At RF level, depletion of DNA2 was shown to result in an accumulation of chicken-foot structures by EM, both during unperturbed growth and under conditions of exogenous replication stress. This phenotype was aggravated upon co-depletion of the helicase RECQ1 [68]. RECQ1 can mediate DNA branch migration, essentially moving the junction point of a chicken-foot intermediate to the tip of the regressed arm that is formed by the nascent leading and lagging strands upon RF reversal. In the wake of the migrating junction point, the unwound nascent strands reanneal to the parental template, which leads to restoration of a canonical three-way RF structure by this non-nucleolytic conversion [121]. DNA2 therefore appears to act in a parallel pathway to RECQ1-mediated branch migration to counteract RF reversal and promote stalled RF recovery in human cells [68]. In unperturbed conditions, DNA2 depletion results in relatively high levels of reversed RFs [68] and post-replicative chromosomal links at mitosis [16]. Together, these observations suggest that DNA2′s role in RF recovery is indispensable for unperturbed replication and directly linked to the most severe cellular phenotypes that have been observed in its absence. Consistent with this notion is the apparent vulnerability of difficult-to-replicate regions of the genome to loss of DNA2. As discussed in Section 2.3.2, DNA2 is needed to properly replicate the centromeric and telomeric DNA in mammalian cells [8,117]. Human DNA2 is enriched at centromeric DNA, and both its helicase and nuclease activity were required for unfettered RF progression and normal deposition of histone variant CENP-A at the centromere. Without DNA2, centromeric replication was associated with replication stress, as evidenced by local activation of the apical checkpoint kinase ATR [117], a potential consequence of loss of centromeric structural integrity [122]. Telomeric DNA is another endogenous source of enhanced RF stalling due to its repetitive DNA sequence, transcriptional activity, and unidirectional mode of replication. In mouse cells, DNA2 haploinsufficiency was accompanied by telomere fragility and loss, consistent with elevated levels of replication stress [8]. Interestingly, an involvement of DNA2 in ameliorating telomere-specific replication stress echoes with observations in cancer cells that use a recombination-based mechanism, alternative lengthening of telomeres (ALT), for telomere maintenance in lieu of telomerase reactivation. Accordingly, ALT was increased after depletion of DNA2 [123]. Since ALT is thought to be initiated by replication stress at telomeres [124], this indicates that loss of DNA2 exacerbates replication stress at telomeres [123]. In unchallenged conditions, the difficult-to-replicate regions of the genome may confer the essential requirement for Dna2^DNA2^ for cell survival.

How does human DNA2 act on stalled RFs? In Section 2.2.1, we have described evidence from studies in yeast suggesting that the Dna2 nuclease and helicase activities cooperate in the processing of stalled RFs to counteract RF reversal. It has been proposed that Dna2 degrades any dissociating nascent DNA, thereby preempting chicken-foot formation [67], and the same may apply to human DNA2. Alternatively, DNA2 could attack a fully-fledged chicken-foot structure after the dissociated leading and lagging strands have annealed with one another. The tip of the regressed arm generated in this way resembles a DSB-end and DNA2 could act on it alone (see Figure 1 and Figure 3) or, as in DSB end-resection (see Section 2.3.1), with a supporting helicase that mediates duplex unwinding. The latter possibility is supported by the finding that DNA2 and WRN are both required for the efficient resumption of DNA synthesis after hydroxyurea-induced RF stalling [68]. It has been proposed that DNA2 and WRN collaborate in the controlled unwinding and degradation of the regressed arm at reversed RFs, and that this restores a three-way configuration that allows DNA synthesis to resume [66,68].

While the precise actions of Dna2^DNA2^ at stalled RFs remain to be determined, it is clear that, in its absence, replication intermediates remain unresolved, and genome duplication incomplete, in both yeast and human cells. The literature to date is consistent with the conjecture that the essential role of human DNA2 is intimately linked to the recovery of stalled RFs, reflecting what has been suggested in yeast [84]. Whether, as proposed in yeast, there is an additional risk of alternative RF restart by unscheduled RDR or other pathways associated with checkpoint activation and cell-cycle arrest remains to be addressed in human cells [30,84].

### 3.2. Controlled RF Processing and Recovery by DNA2 vs. Hyperresection of Unprotected Forks

In addition to its role in the controlled processing and recovery of stalled RFs, human DNA2 has been implicated in the extensive degradation of nascent DNA under certain conditions. When fork progression is blocked over long periods of time by incubating cells in the presence of hydroxyurea for several hours, DNA2 can degrade long stretches of newly synthesized DNA equivalent to several kilobases at stalled RFs [68,125]. Using DNA fiber analysis, it has been shown that this reaction requires the nuclease activity of DNA2 and the helicase activity of WRN, while the DNA2 helicase activity was dispensable [68]. This is in contrast to Dna2^DNA2′^s RF recovery function, which does depend on its nuclease and helicase activities [16,74,117].

DNA2-dependent hyperresection has also been observed at de-protected stalled RFs in cells missing one of a group of proteins that have been shown to control nuclease access at stalled replication intermediates. Accordingly, DNA2 has been shown to drive excessive nascent-strand degradation in the absence of BRCA2, RIF1, BOD1L, FANCD2, ABRO1, and 53BP1 [126,127,128,129,130,131,132]. The pathological nature of stalled RF hyperresection is exemplified in a study showing that depletion of DNA2 suppresses the sensitivity of cells lacking fork-protection factor FANCD2 to replication-blocking DNA cross-linking agents [126]. Other end-resection nucleases catalyze similar reactions at de-protected RFs but appear to be more selective. For example, MRE11 and EXO1 degrade stalled RFs in the absence of BRCA2, yet, not upon loss of ABRO1 [128,133]. The basis for the versatility of DNA2, and its ability to degrade persistently stalled RFs in the presence of the full fork-protection machinery, in contrast to MRE11 and EXO1 [68], is currently unclear. The substrate for DNA2-dependent hyperresection appears to be the chicken-foot structure. The formation of chicken-foot intermediates at stalled RFs in human cells is a complex and active process that requires a host of factors such as RAD51 (in a recombination-independent role) and the SNF2-family fork-remodeling enzymes SMARCAL1, HLTF, and ZRANB3 [134]. If any of these factors are missing, RF reversal and uncontrolled hyperresection of stalled forks by DNA2 is suppressed [68,132].

Importantly, while fork hyperresection arises from the engagement of DNA2 with stalled replication intermediates, it is not a direct reflection of its RF recovery function. If this were the case, DNA2 depletion should attenuate activation of the S phase checkpoint kinase CHK1, which requires the generation of ssDNA. While such an attenuation has been observed upon depletion of DNA2, this only occurs after prolonged replication stress, probably because persistently stalled RFs disintegrate into DSBs, and DSB processing is slowed in the absence of DNA2′s end-resection activity (see Section 2.3.1). Under more physiological conditions, CHK1 signaling is elevated upon depletion or loss of DNA2, consistent with the notion that DNA2 offsets replication stress and checkpoint signaling at stalled RFs [16,117]. Moreover, transient replication stress does not lead to the type of fork hyperresection seen after prolonged fork stalling. However, transient replication stress is sufficient to create a requirement for DNA2 to mediate RF recovery [68]. This suggests that nascent-strand degradation by DNA2 at stalled RFs under physiological conditions does not extend over kilobases. Rather, controlled and limited fork processing by DNA2 is the pathway to RF recovery and replication completion.

## 4. Links between DNA2′s Critical Role in DNA Replication and Human Disease

*DNA2* mutations have been implicated in genetic diseases. Germline mutations in one allele of *DNA2* are associated with mitochondrial myopathy, while biallelic mutations give rise to Seckel syndrome. In addition, DNA2 over-expression has been observed in cancer and the protein is emerging as a potential target for anti-cancer therapy.

### 4.1. DNA2 in Human Genetic Diseases

#### 4.1.1. Mitochondrial Myopathy

**Figure 5:**
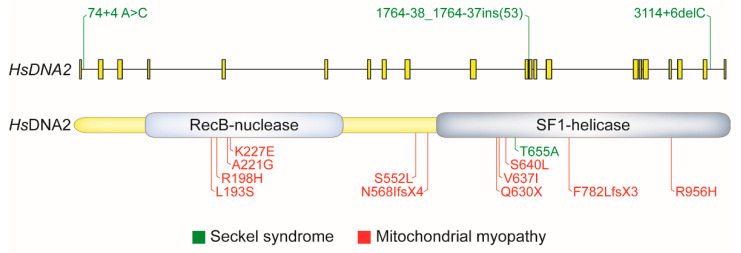
*DNA2* mutation in human genetic disease. Diagram showing the location and type of *DNA2* mutations in mitochondrial myopathy and Seckel syndrome. Gene structure showing introns and exons (**top**) and DNA2 protein domains (**bottom**) are depicted (SF1, superfamily 1). Mutations occurring in introns are marked on the gene, whereas coding mutations are marked on the protein with the resulting amino acid changes indicated.

Adult-onset mitochondrial myopathy is a genetic disease characterized by mtDNA instability and a progressive decline in muscle strength. Heterozygous missense mutations in *DNA2* have been identified in previously genetically uncharacterized patients, supporting a proposed role of DNA2 in maintaining the mitochondrial genome (see Section 2.3.2). Muscle tissue from each patient showed a range of mtDNA deletions [12]. Heterozygous *DNA2* truncating mutations have been linked to an early onset of myopathy, observed in patients younger than two years [14,135,136]. It currently is not clear whether these mutations result in truncated versions of the protein, or whether transcripts are degraded by nonsense-mediated decay leading to complete loss of the protein. Accordingly, DNA2 haploinsufficiency or deleterious actions of truncated versions of DNA2 could contribute to early disease onset. The identified missense mutations map to the helicase and nuclease domains, pointing to the importance of both enzymatic activities of DNA2 at normal capacity for mtDNA maintenance (Figure 5). Purified mutant proteins with single point mutations showed reduced nuclease and helicase activities, and, in one case, reduced nuclease but enhanced helicase activity [12]. Based on other in vitro experiments, it has been suggested that DNA2 may stimulate replication progression and/or RNA primer and DNA-flap removal during replication and the repair of oxidative damage in mitochondria [118]. The precise role of DNA2 and specific requirements for its helicase and nuclease activities for mitochondrial stability remain to be experimentally determined.

Replication of mtDNA is mediated by a set of nuclear-encoded proteins. Interestingly, autosomal dominant mutations affecting key components of this mtDNA replication machinery, including Pol γ and Twinkle have been linked to mitochondrial myopathy [137,138]. Disease-causing mutations are also found in genes affecting replication progression by maintaining mitochondrial dNTP pools [137]. Modeling of mitochondrial myopathy-causing mutations within Twinkle and Pol γ demonstrated that mtDNA deletions are associated with stalled replication intermediates [139,140]. As mentioned above, DNA2 localizes to mitochondria, where it accumulates further under replication-stress conditions [118,119]. Although differences exist between nuclear and mitochondrial DNA replication, chicken-foot structures such as those counteracted by DNA2 in the nucleus have been detected within mtDNA after induction of replication stress [141]. In light of the intimate links between mtDNA replication progression, mtDNA deletion, and mitochondrial myopathy, it is tempting to speculate that DNA2 might mediate RF recovery in mitochondria in much the same way as in the nucleus, and that perturbations of this function result in mtDNA deletions and mitochondrial disease.

#### 4.1.2. Seckel Syndrome

Seckel syndrome is an autosomal recessive disorder characterized by intrauterine growth retardation, low birth weight, dwarfism, and microcephaly [142]. Several Seckel syndrome patients with causative mutations in *DNA2* have been described [10,11]. Three out of the four mapped mutations are intronic mutations that cause a reduction in the levels of wild-type DNA2 and/or expression of incorrectly spliced transcripts (Figure 5). Interestingly, patients with mitochondrial myopathy, who are predicted to have reduced levels of wild-type DNA2 protein, did not show Seckel-like features [136]. Further analyses are required to determine whether the truncated DNA2 transcripts in various patients are stably expressed and how levels of residual wild-type DNA2 may relate to these distinct disorders. Among *DNA2*-Seckel patients, an individual with an intronic mutation in one allele, and a helicase-domain mutation near the ATP-binding site (DNA2 T665A) in the other, has been identified. This patient presented with the most severe reduction in head circumference and body height [11]. The DNA2 T665A mutation can be expected to have a severe and specific effect on the ATPase/helicase activity, providing a tentative link between Seckel syndrome and RF recovery, a function that clearly requires DNA2 helicase activity in vivo.

Like mitochondrial myopathy, Seckel syndrome is genetically heterogeneous and disease mutations affect groups of genes with roles in DNA replication/repair (*ATR*, *DNA2*, *CTIP*, *NSMCE2*, and *TRAIP*) and segregation of the replicated genome into daughter cells (*CEP63*, *CEP152*, *CENPJ*, *NIN*, and *NSMCE2*). When these processes are compromised, cell proliferation is severely hampered, providing an explanation for growth defects associated with Seckel syndrome. Some Seckel syndrome genes suggest striking parallels to DNA2′s function in RF recovery and replication completion. For example, ubiquitin ligase TRAIP promotes the completion of chromosomal replication and segregation by mediating the clearance of replication obstacles in S phase and removal of the replication machinery from sister chromatids in mitosis [143,144]. Similarly, DONSON, mutated in Seckel-like disease, plays a key role in RF stability and replication completion [145,146,147]. By extension, impaired RF recovery, incomplete DNA replication, and the resulting cell-proliferation defects in the absence of fully functional DNA2 [16,68,117] provide a potential explanation for the involvement of *DNA2* mutations in primordial dwarfism.

### 4.2. DNA2 as a Potential Anti-Cancer Target

From yeast to human, Dna2^DNA2^ is required to drive chromosomal replication to completion. Consequently, when Dna2^DNA2^ is impaired, depleted, or lost, underreplication ensues and results in the type of chromosome segregation problems and chromosomal aberrations frequently seen in cancer cells [15,16,17,28,45,84,117,119]. *DNA2*^+/−^ mice exhibit genome instability and a higher incidence of cancer, suggesting that *DNA2* haploinsufficiency drives malignant transformation [8]. Significant levels of *DNA2* mutations have been seen in some cancer types in human (reviewed in [148]). Whether these mutations drive genome instability and contribute to tumorigenesis is currently unclear and will require a better understanding of the impact each individual mutation might have on *DNA2* function. On the other hand, *DNA2* amplification and over-expression has been reported for a range of cancers including breast, pancreatic, and colorectal cancer [17,21,148,149,150]. This suggests that DNA2 could play a role in supporting cancer-cell proliferation, most likely through its ability to offset replication stress.

#### 4.2.1. DNA2 Promotes the Survival of Cancer Cells with Elevated Levels of Replication Stress

Cancer cells experience greater levels of replication stress compared to healthy cells. Oncogene activation drives cells into S phase with abnormal replication timing, imbalances in origin of replication activity, increased conflicts between replication and transcription, and inappropriate dNTP pools. As a consequence, RF progression is chronically impaired, and genome damage arising from chromosome underreplication is frequent [151,152,153]. Increased expression of *DNA2* could therefore reflect an adaptation of cancer cells that helps them cope with elevated levels of endogenous replication stress and increased requirements for RF recovery and DNA repair. In support of this notion, elevated DNA2 levels have been shown to correlate with a poor prognosis for breast cancer patients and stemness of cancerous cells in colorectal cancer [149,150]. Experimentally, DNA2 has been shown to promote cancer cell survival following overexpression of H-RAS or Cyclin E, suggesting a role for DNA2 in overcoming oncogene-induced replication stress [17].

#### 4.2.2. Targeting DNA2 as an Anti-Cancer Strategy

There is an increasing realization that endogenous replication stress represents an Achilles heel in cancer. While intrinsic replication stress may fuel tumorigenesis through the accumulation of cancer-promoting mutations, there is also the potential of exploiting it as a weapon against cancer cells. Hence, depriving cancer cells of key replication stress response factors may affect catastrophic genome damage and cell-cycle arrest. The potential for achieving this type of clinical synthetic lethality is exemplified by inhibitors targeting the replication stress response kinases ATR and CHK1, which have reached clinical-trial stage [154]. Enzyme effectors of the replication stress response and RF recovery, including DNA2, may be equally well-suited drug targets for anti-cancer therapy [155].

Working on this premise, two small-molecule DNA2 inhibitors (DNA2i) have been identified [20,21]. NSC-105808 (6-amino-7-bromoquinoline-5,8-dione) was shown to inhibit the nuclease activity of DNA2 and restrict cancer cell proliferation to a greater extent compared to non-transformed control cells [21]. Various cell types were sensitized to NSC-105808 through oncogene expression (H-RAS, K-RAS, or human papillomavirus E6 and E7), suggesting that DNA2i may have the potential to act in a broad range of cancers characterized by endogenous replication stress. Inhibitor studies have also focused on combining DNA2i with treatments that further perturb RF progression. Inhibitor C5 (4-hydroxy-8-nitroquinoline-3-carboxylic acid) was demonstrated to interfere with DNA2′s ability to bind DNA, thus impacting both the helicase and nuclease activity of DNA2 [20]. Co-treatment of cells with C5 and exogenous replication stress acted to synergistically reduce cell viability. Similarly, C5 and PARP inhibitors acted synergistically to inhibit cancer cell growth [20]. Poly(ADP-ribosyl)ation mediated by PARPs is a key posttranslational modification for DNA repair and the recovery of stalled RFs [156,157,158]. Synergy between DNA2 and PARP inhibitors would be consistent with a model where DNA2 counteracts RF reversal, and if this is inhibited, PARP becomes critical for controlled chicken-foot formation, processing, and RF restart.

The identification of small-molecule inhibitors of DNA2 is an exciting development [20,21]. Although DNA2 is essential in healthy cells, the available data suggests that there may be a therapeutic window large enough to titrate DNA2i for selective killing of the more vulnerable cancer cells with elevated replication stress levels, while sparing healthy cells. In addition, certain cancers may be further sensitized to DNA2 inhibition on account of pre-existing mutations. This is exemplified by results showing greater efficacy of DNA2i in killing cells harboring a gain-of-function mutant of p53 expressed in many cancers [159]. These findings expand the potential of DNA2 inhibition for cancer therapy and may help to stratify patients most likely to benefit from DNA2i treatment. Based on the functional interplay between Dna2 and Holliday junction resolvase Yen1 in budding yeast [74,75], it would be interesting to determine whether cancer cells that escape DNA2i might be susceptible to targeted inhibition of one of the junction-resolving nucleases known to target replication intermediates in human cells [78].

## 5. Concluding Remarks and Future Perspective

The Dna2^DNA2^ nuclease-helicase has been implicated in a variety of DNA metabolic processes including OFP, DSB end-resection, RF recovery, and the pathological hyperresection of de-protected stalled RFs. It follows that the interpretation of cellular phenotypes and human disease syndromes associated with loss of Dna2^DNA2^ function is inherently difficult. Each observed defect could potentially have complex molecular underpinnings involving multiple pathways. However, there are clearly common themes of incomplete chromosomal replication, replication-stress sensitivity, and ineffective recovery of stalled RFs associated with perturbations of Dna2^DNA2^ across organisms. The involvement of DNA2 in mitochondrial myopathy and Seckel syndrome are consistent with a defect in replication progression within the mitochondrial and nuclear genomes, and small-molecule DNA2i successfully impair RF recovery to generate synthetic lethality in cancer cells with endogenous replication stress. Thus, despite the complexity surrounding the multi-faceted capabilities of Dna2^DNA2^, a unifying view is emerging around a central role of Dna2^DNA2^ in the processing and recovery of stalled RFs. This role is essential in yeast and provides a plausible framework to rationalize why DNA2 is indispensable for mammalian cell proliferation and human health.

Is it all about RFs? Certainly not. Dna2^DNA2^ contributes to chromosome break repair by integrating with the DSB end-resection machinery. At least in yeast, a strong case has been made for its participation in OFP. Although a major role for Dna2^DNA2^ in OFP has not transpired in vitro or in vivo, its ability to degrade RPA-covered ssDNA expands the repertoire of substrates that can be processed in cells during lagging-strand maturation. Similarly, the versatile Dna2^DNA2^ nuclease-helicase is likely to be employed in further DNA repair processes. Nonetheless, the most critical activity of Dna2^DNA2^ appears to be at stalled RFs. In the future, it will be important to define more precisely the actions of Dna2^DNA2^ that counteract RF reversal, resolve fully-fledged chicken-foot intermediates, allow stalled RFs to resume DNA synthesis, limit recombination-based restart, and/or promote RF fusion. These processes are critical for the completion of chromosomal replication across species. A better mechanistic understanding of Dna2^DNA2^ and its interplay with other factors will be key to inform the ongoing efforts to develop DNA2 as a target for anti-cancer therapy.

## Abbreviations

ALT	alternative lengthening of telomeres
BIR	break-induced replication
D-loop	displacement loop
DNA2i	DNA2 inhibitors
dNTP	deoxyribonucleotide triphosphate
DSB	DNA double-strand break
dsDNA	double-stranded DNA
EM	electron microscopy
G4	G-quadruplex DNA
mtDNA	mitochondrial DNA
nt	nucleotide(s)
OFP	Okazaki fragment processing
Pol	DNA polymerase
RDR	recombination-dependent replication
RF	DNA replication fork
SF1	superfamily 1
ssDNA	single-stranded DNA
